# Ontogenetic Trajectories of Chimpanzee Social Play: Similarities with Humans

**DOI:** 10.1371/journal.pone.0027344

**Published:** 2011-11-16

**Authors:** Giada Cordoni, Elisabetta Palagi

**Affiliations:** 1 Natural History Museum, University of Pisa, Pisa, Italy; 2 Vademecos, Biology Professional Association, Viareggio, Italy; 3 Unit of Cognitive Primatology and Primate Center, Institute of Cognitive Sciences and Technologies, Consiglio Nazionale delle Ricerche (CNR), Roma, Italy; University of Lethbridge, Canada

## Abstract

Social play, a widespread phenomenon in mammals, is a multifunctional behavior, which can have many different roles according to species, sex, age, relationship quality between playmates, group membership, context, and habitat. Play joins and cuts across a variety of disciplines leading directly to inquiries relating to individual developmental changes and species adaptation, thus the importance of comparative studies appears evident. Here, we aim at proposing a possible ontogenetic pathway of chimpanzee play (*Pan troglodytes*) and contrast our data with those of human play. Chimpanzee play shows a number of changes from infancy to juvenility. Particularly, solitary and social play follows different developmental trajectories. While solitary play peaks in infancy, social play does not show any quantitative variation between infancy and juvenility but shows a strong qualitative variation in complexity, asymmetry, and playmate choice. Like laughter in humans, the playful expressions in chimpanzees (at the different age phases) seem to have a role in advertising cooperative dispositions and intentions thus increasing the likelihood of engaging in solid social relationships. In conclusion, in chimpanzees, as in humans, both play behavior and the signals that accompany play serve multiple functions according to the different age phases.

## Introduction

Due to its multifunctional and complex nature, play is one of the most difficult behaviors to study [1]–[3]. Play apparently is difficult to define and definitions vary widely among researchers. For instance, in humans, there is considerable confusion surrounding the definition of play. In fact, in the child development literature, a variety of children's social and non-social behaviors are grouped under the term “play” [2]. For clarity, in this paper we will use ethological and sociobiological definition of play [2], [4], [5].

Ethologists and sociobiologists often define play as all activity, which has no clear, immediate benefits. Frequently, play seems to involve an array of motor patterns, both typical of serious functional contexts (e.g. agonistic, antipredatory, and mating behavior) [6]–[8] as well as playful actions [9]. However, the main difference between playful and serious contexts is not in the actual behavioral patterns, but how they are performed [10], [11]. Burghardt [1] listed five criteria that a behavior must follow to be considered play; a playful behavior must be incompletely functional, rewarding/voluntary, structurally or temporally modified, performed in a repeated manner, and initiated in a relaxed context [1].

Physical activity play is one of the most common forms of play. It typically implies locomotor-rotational/acrobatic (LR-play) patterns that can be carried out both solitarily and socially. Rough and tumble play (R&T), is characterized by fighting elements (performed in non-serious way), which involve more than one player [12]–[16]. Here, we focus on social, physical activity involving both LR-play and R&T.

Social play is widespread in mammals [1]. This behavior has different functions according to species, sex, age, relationship quality between playmates, group membership, context, and habitat [17]–[22]. Clearly then, play joins and cuts across a variety of disciplines. It leads directly to inquiries connecting individual development with species adaptation. It is not surprising that comparative studies of play behavior can make contributions to a wide variety of fields [23].

Social play is a fundamental component of the behavioral repertoire of the youngsters of many species of mammals, including humans, and its developmental trajectories (onset, peak, and offset) have evolved in concert with the extension of the immaturity period [20], [24]–[27]. Social play is first experienced between mother and offspring. Many good examples come from human and non-human primates [28], [29]. Peek-a-boo, a typical mother-child game, also occurs in the great apes as do other locomotor activities that involve bouncing, throwing, and swinging infants [4]. Interactions with their mothers represent for infants a good starting-point to learn how to manage play sessions (fine-tuning) that will later be transferred and fully developed in peer-peer interactions [4], [30], [31]. The quality and quantity of mother-infant play seem to predict the quality and frequency of infants' play with peers [2], [32]. However, peer-peer play can remediate deficits in mother-infant play and contribute sufficiently to normal social development in monkeys [33], great apes [34], and children [35]–[37]. This suggests that infant-infant play is important for acquiring social competence and developing affinitive bonds.

Primate social play in older immature subjects also functions to establish a dominance order among individuals. Individuals acquire information on the strength and weakness of group members by engaging in R&T (humans [38], [39]; chimpanzees, [40]).

In chimpanzees social play serves different functions at different ages (Hypothesis 1) [11], [14], [19], [41]. According to Hypothesis 1, chimpanzee play should vary in modality (e.g. asymmetry in R&T, competition), complexity (variability in the play patterns used), selectivity of playmates (e.g. age selectivity), and frequency as a function of individual age classes (Prediction 1).

One of the most important reasons for studying play in chimpanzees is to shed light on the biological roots of human behavior. Due to their phylogenetic closeness and prolonged immaturity phase [1], [3], [20], [23], chimpanzees and humans have similar developmental pathways for play (Hypothesis 2). Accordingly, both species should show similar play parameters (e.g., modality, complexity, selectivity of playmates, and frequency) across age-classes (Prediction 2).

Specific facial displays (the relaxed open-mouth display or play face is usually associated with pant-like vocalizations) often accompany play sessions [11], [13], [42] In primate species, facial displays are a fundamental key for successfully managing play bouts [43]. Playful facial signals have to be considered as an integral part of play behavior development [44]. Great apes perform playful facial displays via two different configurations; play face (PF), where the mouth is open with only the lower teeth exposed, and full play face (FPF), where the mouth is opened with upper and lower teeth exposed. Some authors contend that the two expressions are used differently in relation to the intensity of play [11], [45], [46]. In humans, laughter, which is a universal expression [47], [48], seems to derive from non-human primate play faces and pant-like vocalization [49]. Recently, an affect-induction hypothesis has been proposed to elucidate the function of human laughter: this expression does not give simple information, but induces a positive influence on the receiver behavior [49]–[54]. Humans and apes smile spontaneously during pleasurable experiences, including visual, auditory, and tactile stimulation. Smiles and play faces, being the expression of positive emotional states, reinforce the behaviors that elicited them in the first place. Such kind of visual reinforcement is essential for learning in infants, when mothers smile at babies to encourage desired behaviors (see [55]–[57] for an extensive review).

Although playful facial configurations can differ across species [8], they may have a common role in signaling non-agonistic intent to a playmate and/or in expressing emotion even when alone [42], [44] (Hypothesis 3). If in chimpanzees play faces are used in a strategic way and function to signal benign intent, they should vary in frequency, timing, and type (PF & FPF) according to the age of playmates (Prediction 3a). If play faces mainly signal internal emotional states of the player [58], they should not necessarily vary across the playmates' ages (Prediction 3b).

## Results

The present study was carried out on two captive groups of *Pan troglodytes* hosted at the ZooParc de Beauval (S. Aignan sur Cher, France) and the Dierenpark Amersfoort (Amersfoort, The Netherlands). The Beauval colony was composed of 2 adult males, 8 adult females, 3 juvenile males, 2 juvenile females, 2 infant males and 2 infant females; the Amersfoort colony was made up of 2 adult males, 9 adult females, 2 juvenile females, 2 infant males and 2 infant females (Table 1).

**Table 1:** The chimpanzee colonies hosted at the ZooParc de Beauval and Dierenpark Amersfoort, respectively.

SUBJECTS (INITIALS)	SEX CLASS	YEARS/AGE CLASS	SIBLINGS RELATIONSHIP	RESIDENCE
Christmas (CR)	Female	6.5/Juvenile	LE's sister	Beauval
Isabel (IS)	Female	5.5/Juvenile		Beauval
Melie (ME)	Female	3.5/Infant		Beauval
Rachel (RA)	Female	1.0/Infant		Beauval
Tsavo (TS)	Male	7.0/Juvenile	BZ's brother	Beauval
Benji (BE)	Male	6.0/Juvenile	MA's brother	Beauval
Leo (LE)	Male	4.0/Juvenile	CR's brother	Beauval
Makury (MA)	Male	2.5/Infant	BE's brother	Beauval
Bazou (BZ)	Male	2.0/Infant	TS's brother	Beauval
Bibi (BI)	Female	7.0/Juvenile	KR's sister	Amersfoort
Chura (CH)	Female	6.0/Juvenile		Amersfoort
Ghafula (GA)	Female	3.5/Infant	IT's sister	Amersfoort
Ituri (IT)	Female	0.5/Infant	GA's sister	Amersfoort
Karibuna (KR)	Male	2.5/Infant	BI's brother	Amersfoort
Kumi (KU)	Male	2.0/Infant		Amersfoort

Via focal animal sampling [59] we collected data on both social and solitary play behavior: Locomotor/Rotational-play (LR-play), Rough&Tumble (R&T), Object play (O-play). See the Methods and Table 2 for the definitions.

**Table 2:** Play behavioral patterns recorded during the observation sessions both at the Beauval colony and the Amersfoort colony.

Locomotor-Rotational play	Initials	Definition
Acrobatic Play	ACP	An animal climbs, jumps, and dangles from supports in its environment (e.g., branches, ropes, etc.) in solitary or social way (animals climb, jump, and dangle together and concurrently often on the same support, B*).
Pirouetting	PIRO	An animal performs rolling over either on the ground or on vertical supports in solitary or social way (animals roll in contact hanging on the same vertical support, B)
Play recovering a thing	PRCO	Animal chases playmate and attempts to grab object carried by it (U*)
Play run	PRUN	Animal runs alone (solitary play) or chases play partner (social play) (U)
Somersault	SO	An animal flips over either on the ground or on vertical supports in solitary or social way (animals flip in contact, B)
**Rough and Tumble play**		
Play bite	PBIT	Animal gently bites playmate (U)
Play brusque rush	PBR	Animal jumps with its four limbs on playmate (U)
Play push	PPS	Animal pushes playmate either with its hands or feet (U)
Play retrieve	PRE	Animal holds playmate to prevent its flight (U)
Play slap	PSL	Animal slaps any part of playmate's body (U)
Play stamping	PST	Animal jumps on the ground (solitary) or on a playmate with its feet (social, U)
**Other Play Patterns**		
Full play face	FPF	Playful facial display: mouth is opened with upperand lower teeth exposed
Object play manipulation	OPM	Animal shakes, dangles, throws, an object of its environment in solitary or social way (when the action is directed to a playmate; the pattern does not imply any kind of contact between the two animals)
Play face	PF	Playful facial display: mouth is opened with only lower teeth exposed
Tickle	TK	An animal contacts the partner's body with its mouth or hands (U)

* B = Bidirectional pattern; U = Unidirectional pattern.

**Predictions 1 & 2:** *There should be age-related changes in the frequency and content of chimpanzee play (**P1**) and strong similarities between chimpanzee and human play development (**P2**)*

### Frequency of play

**Figure 1:**
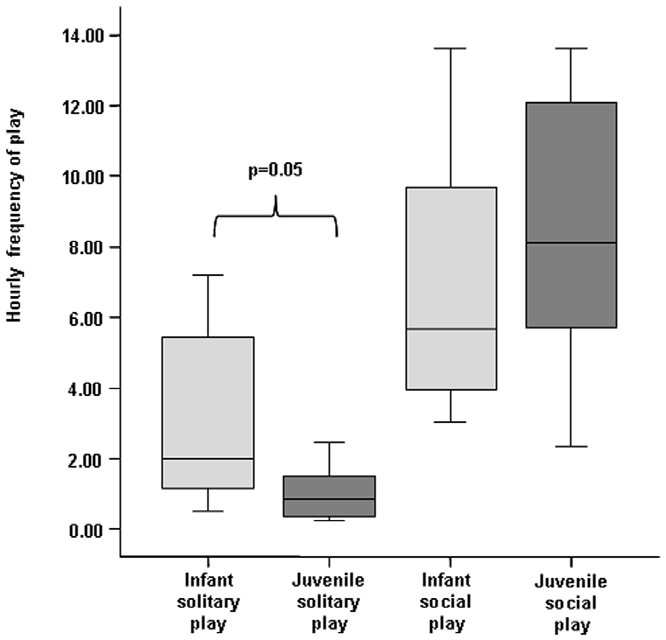
Hourly frequency of solitary and social play, respectively, in relation to age-class (infants and juveniles). Solid horizontal lines indicate medians; length of the boxes corresponds to inter-quartile range; thin horizontal lines indicate range of observed values. Only significant results are reported.

Social play (LR−play+R&T) was significantly more frequent than solitary play (O−play+LR−play) both in infants (I) (Exact Wilcoxon's T = 0, ties = 0, n = 8, P = 0.008) and juveniles (J) (Exact Wilcoxon's T = 0, ties = 0, n = 7, P = 0.016). However, infants performed solitary play more often than juveniles (Exact Mann-Whitney U = 11.0, n_I_ = 8, n_J_ = 7, P = 0.050); social play did not differ between the two age categories (Exact Mann-Whitney U = 22.5, n_I_ = 8, n_J_ = 7, P = 0.558) (Figure 1). Solitary play accounted for 29.27%±5.9SE of all play behavior in infants and for 14.45%±5.2SE, in juveniles.

### Playmate choice

**Figure 2:**
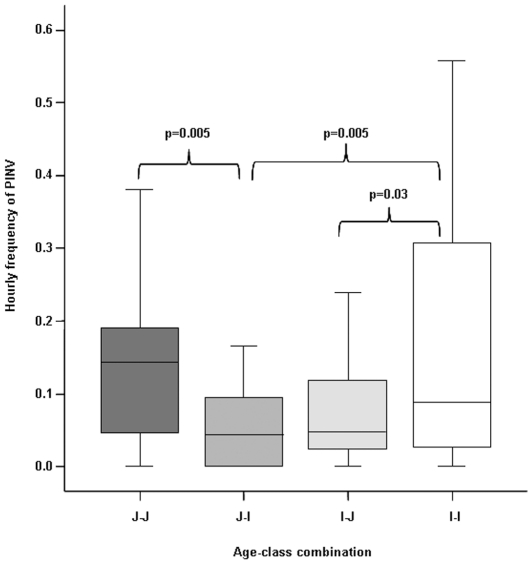
Hourly frequency of play invitation directionality (PINV) in relation to the different age-class combinations: juvenile-juvenile (J-J), juvenile-infant (J-I), infant-juvenile (I-J) and infant-infant (I-I). Solid horizontal lines indicate medians; length of the boxes corresponds to inter-quartile range; thin horizontal lines indicate range of observed values. Only significant results are reported.

The age of the playmates (see Methods for definition) affected the play invitation (PINV, see Table 2 for definition) distribution (ANOVA randomization F = 3.756, n_JJ_ = 21, n_JI_ = 23, n_IJ_ = 23, n_II_ = 24, P = 0.006) (Figure 2). The post-hoc test revealed the following significant differences: PINV_JJ_>PINV_JI_ (randomization test for two independent samples t = −2.9, P = 0.005), PINV_II_>PINV_IJ_ (t = 2.02, P = 0.030), and PINV_II_>PINV_JI_ (t = 2.50, P = 0.005).

### Play modality

**Figure 3:**
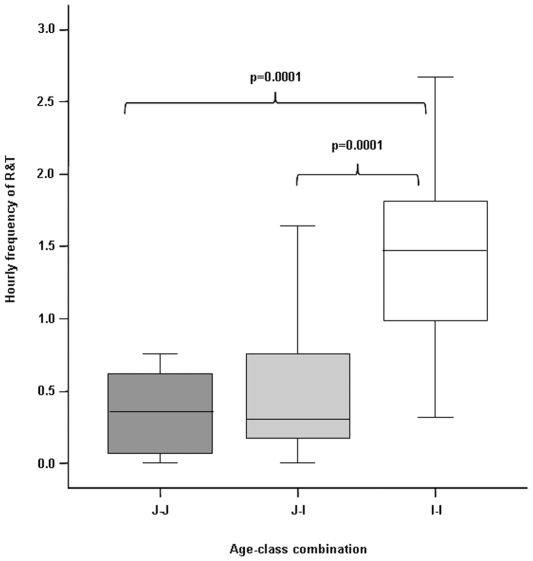
Hourly frequency of rough-and-tumble (R&T) as a function of the ages of players: juvenile-juvenile (J-J), juvenile-infant (J-I), and infant-infant (I-I). Solid horizontal lines indicate medians; length of the boxes corresponds to inter-quartile range; thin horizontal lines indicate range of observed values. Only significant results are reported.

R&T distribution was also affected by the age of playmates (ANOVA randomization F = 17.95, n_JJ_ = 10, n_JI_ = 24, n_II_ = 11, P = 0.000). Post-hoc tests revealed that I-I play levels were significantly higher than those of I-J and J-J (randomization test for two independent samples: J-J vs J-I: t = 0.666, P = 0.518; J-J vs I-I: t = −4.152, P = 0.000; J-I vs I-I: t = 5.077, P = 0.000) (Figure 3). On the other hand, no difference was found in the LR-play distribution according to the age of playmates (ANOVA randomization F = 0.99, n_JJ_ = 10, n_JI_ = 24, n_II_ = 11, P = 0.379). We limited these analyses to those dyads performing at least two play sessions.

### Play asymmetry

We defined an *Asymmetry Index* (AI) to quantify the level of asymmetry that characterized a play session. The index was defined as the number of unidirectional patterns performed by A minus the number of unidirectional patterns performed by B on the total number of patterns (unidirectional and bidirectional, see Methods for definitions) forming the session (session length). By subtracting the number of unidirectional patterns performed by B from those performed by A, we obtained an estimate of difference between unidirectional patterns which was independent of the length of the play session. However, in order to be more conservative for the calculation of the asymmetry index we considered only the dyads performing at least five play sessions, each one composed of at least ten unidirectional play patterns. For each dyad the median of the index was calculated and entered into the analysis.

The mismatched (I-J) and matched (I-I and J-J) dyads did not differ in the median values of AI (Two independent randomization test t = 0.623, n_mismatch_ = 13, n_match_ = 19, P = 0.560).

Focusing on the matched dyads (I-I and J-J), we found that the AI values of play between infants were higher than those between juveniles (Two independent randomization test t = 1.902, n_II_ = 10, n_JJ_ = 9, P = 0.040).

### Play complexity

In order to estimate the variability of play patterns forming a single play session we defined the *Play Complexity Index* (PCI) as the number of different types of play patterns performed by playmates within a single session on the total number of patterns forming that session. To be more conservative, we calculated the PCI by considering only the dyads performing at least five play sessions each one composed by at least ten play patterns (both unidirectional and bidirectional). For each dyad the median of the index was calculated and entered into the analysis.

**Figure 4:**
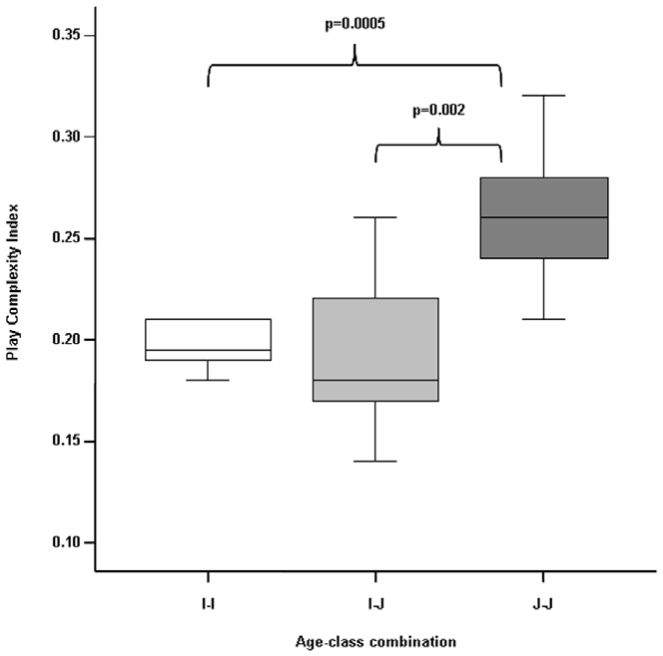
Play Complexity Index as a function of the ages of players: juvenile-juvenile (J-J), juvenile-infant (J-I), and infant-infant (I-I). Solid horizontal lines indicate medians; length of the boxes corresponds to inter-quartile range; thin horizontal lines indicate range of observed values. Only significant results are reported.

The PCI differed across the age-class combination (ANOVA randomization F = 10.497, n_JJ_ = 10 dyads, n_IJ_ = 17 dyads, n_II_ = 10 dyads, P = 0.000). Post-hoc tests revealed a significant difference between J-J and I-J dyads (JJ>IJ: two independent randomization test t = −3.534, n_JJ_ = 10 dyads, n_IJ_ = 17 dyads, P = 0.002) and between J-J and I-I dyads (JJ>II: t = −4.156, n_II_ = 10 dyads, n_JJ_ = 10 dyads, P = 0.001); no significant difference was found between I-I and I-J dyads (t = 0.963, n_II_ = 10, n_IJ_ = 17, P = 0.368) (Figure 4).

**Prediction 3a:** *If in chimpanzees play faces are used in a strategic way and function to signal benign intent, they should vary in frequency, timing, and type (PF & FPF) according to the age of playmates.*

**Prediction 3b:** *If play faces mainly signal internal emotional states of the player, they should not necessarily vary across the playmates' ages.*

### Play signal frequency

Infants and juveniles did not differ in the overall frequency of play signals (PF+FPF per play session) (Exact Mann Whitney U = 26, n_I_ = 8, n_J_ = 7, P = 0.867). Both infants and juveniles performed more facial displays (PF+FPF per play session) during social rather than solitary play (I, Exact Wilcoxon's T = 0, ties = 0, n = 8, P = 0.008; J, Exact Wilcoxon's T = 1, ties = 0, n = 7, P = 0.03).

### Play signal timing

We calculated how many times a playful facial display was performed at the beginning or in the middle of each play session in order to evaluate if the signal was used to initiate or maintain the session. Immature chimpanzees displayed play signals significantly more often to maintain rather than to initiate a social play session (Randomization paired t test: t = 6.715, n = 42, P = 0.000). The result did not change even considering all the possible age-class combinations separately (Randomization paired t test I-I: t = 3.476, n_II_ = 12 dyads, P = 0.000; I-J: t = 4.843, n_IJ_ = 20 dyads, P = 0.000; J-J: t = 5.305, n_JJ_ = 11 dyads, P = 0.003). We entered into the analysis only those sessions formed by at least three play patterns.

### Preferential use of different play signals

The *Play Signal Index* (PSI) was defined to analyze the preferential use of the variants of playful facial displays (PF or FPF) with respect to the total amount of playful facial signals performed. The index was calculated as follows [(PF-FPF)/(PF+FPF)] as suggested by Palagi [58]. The PSI can be either positive or negative depending on the relative amount of facial expressions performed. Values can vary from −1 (only FPF performed) to +1 (only PF performed). If the value of PSI is 0, PF and FPF are performed with the same frequency.

To analyze the use of the variants of playful facial displays (PF and FPF) as a function of the age of the performers, we compared the *Play Signal Index* (PSI) between infants and juveniles. The PSI did not differ between the two age classes (Exact Mann Whitney U = 23, n_I_ = 8, n_J_ = 7, P = 0.588; mean infant PSI = 0.32±0.11SE, mean juvenile PSI = 0.33±0.12SE).

**Figure 5:**
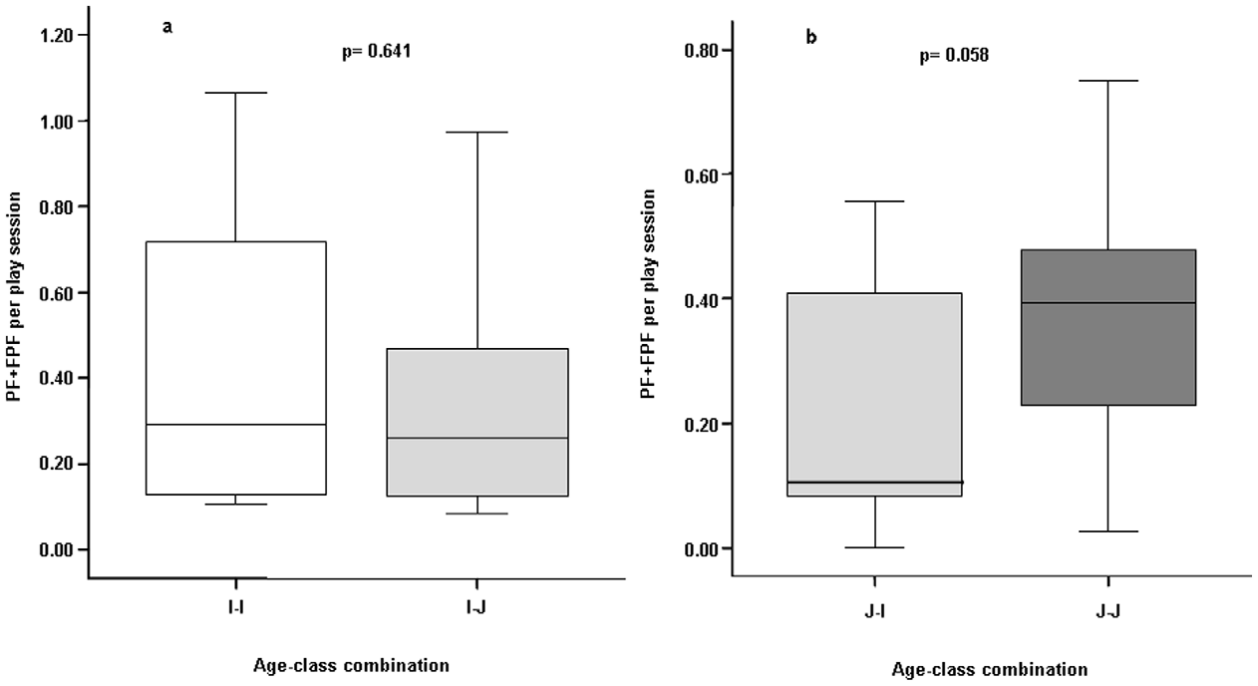
Playful facial displays (Play Face+Full Play Face) *per* play session performed by infants towards other infants (I-I) and juveniles (I-J) (a) and by juveniles towards infants (J-I) and other juveniles (J-J) (b). Solid horizontal lines indicate medians; length of the boxes corresponds to inter-quartile range; thin horizontal lines indicate range of observed values.

Infants directed play signals (PF+FPF per play session) towards peers and juveniles with comparable levels (Exact Wilcoxon's T = 14, ties = 0, n = 8, P = 0.641) (Figure 5a); whereas, juveniles tended to direct play signals towards other juveniles more than towards infants (T = 2, ties = 0, n = 7, P = 0.058) (Figure 5b).

### Play signal and play asymmetry

No correlation was found between the median values of playful facial display per play session and the median values of asymmetry index during play session involving mismatched dyads (J-I) (Correlation via randomization r = −0.331; n = 13, P = 0.242). On the contrary, there was a positive correlation between the median values of playful facial display per play session and the median values of asymmetry index during play session involving matched dyads (I-I and J-J) (r = 0.72; n = 19, P = 0.009). Testing the I-I and J-J dyads, separately, we found a significant positive correlation in the former (Correlation via randomization r = 0.735; n = 10, P = 0.042) and no correlation in the latter (r = 0.467; n = 9, P = 0.194).

## Discussion

As it occurs in humans, play in chimpanzees varied according to the age of the players in relation to different parameters such as modality, complexity, selectivity of playmates, and frequency (Prediction 1 and 2 supported, Table 3). Play signals did not vary in terms of frequency, timing and type across the ages of playmates, even though we found interesting results indicating that both infants and juveniles perform playful facial displays in a strategic way (Prediction 3a partially supported). Moreover, we found that playful expressions were performed even when chimpanzees played alone, thus suggesting that PF and FPF may be directly linked to the emotional state of the sender (Prediction 3b supported).

**Table 3:** Summary of the main comparing aspects of play across the two species, *Pan troglodytes* and *Homo sapiens*

CHIMPANZEES	HUMANS	WHAT ABOUT PREDICTION 2?
Solitary play frequency is higher in motor independent infants than in juveniles (quantitative variation)	Solitary play frequency is higher in kindergarten-aged children than in preschooler ones (quantitative variation) [60]–[62]	supported
Social play (Locomotor-Rotational and Rough-&-Tumble Play, R&T) is uniformly widespread from infancy to juvenility (no quantitative variation). However, social play shows qualitative variation during this transitional phase	Social play frequency (Locomotor-Rotational and Rough-&-Tumble Play, R&T) increases during the transition from kindergarten- to preschooler-aged children (quantitative variation) [2]	not supported
R&T is uniformly distributed from infancy to juvenility (no quantitative variation)	R&T is uniformly distributed in children till 7 years of age (no quantitative variation) [63]	supported
Presence of selectivity for play partners (peer preference)	Presence of selectivity for play partner (peer preference) [3], [31]	supported
In infants, R&T is more frequent than in juveniles (quantitative variation)	No quantitative data are available for a direct comparison [3], [16]	no data available for comparison
Juvenile social play is more complex and innovative (behavioural flexibility for new social challenges)	Adolescent social play is more innovative (behavioural flexibility for new social challenges) [20]	supported
Play asymmetry is more common in infants than in juveniles (less clear-cut relationships or less social competence?)	No quantitative data are available for a direct age comparison [2]. Nevertheless, more balanced play sessions are present when individuals have clear social dominance relationships, that is since early adolescence [20]	no data available for comparison
Both in infants and juveniles playful facial expressions are two times more frequent during social than solitary play (interactive function)	In infants and children, social contexts facilitate laughter. Indeed, laughter bouts are 30 times more likely to occur when individuals are interacting with conspecifics than when alone [57]	supported
In infants, a correlation is present between play asymmetry and playful facial displays; juveniles selectively direct their play faces to other juveniles (signaling a benign intent during potentially ambiguous situations, e.g. retroactive and meta-communicative function)	Although no quantitative data are available for a direct age comparison [57], in infants and children laughter induces a positive influence on the receiver behavior [52]–[54]	no data available for comparison

In humans, the three types of physical activity play (rhythmic stereotypies, exercise play, R&T) show three successive peaks, thus reflecting different functions for these different forms of play [16] (Table 3). Our finding on solitary play is in agreement with data coming from children. Even though parallels across ages of different species have to be taken cautiously, there is a marked overlapping between the percentage of solitary play in infant (29.27%±5.9SE) and juvenile chimpanzees (14.45%±5.2SE) with those of preschoolers (0–3 yrs; 17%–23%) and kindergarten-aged children (3–6 yrs; 17%), respectively [60]–[62] (Prediction 2 supported). Such overlapping seems to disappear when we consider social play. In humans, the transition from solitary to social play occurs during the preschool period [2]; whereas, in chimpanzees social play constantly covers a wide time-window, from infancy (0–3 years) to juvenility (4–7 years). However, if within social play we consider R&T, there are striking similarities between humans and chimpanzees (Prediction 2 supported). Consistent with our findings, Scott and Panksepp [63] demonstrated that R&T play is at a plateau until children are at least 7-years old.

Play effectiveness in the development of social skills often means a choice of an appropriate playmate [13]. Even though studies of children's social play rarely focused on the effects of age on partner choice, it seems that human and non-human primates show selectivity for peers, especially in terms of strength/size matching (Prediction 2 supported, see Table 3) [3], [31]. In humans, for example, this is true also for 9-month-old babies, who show peer preference when they watch movies of same-age infants [64]. In chimpanzees, the selectivity for play partner choice may be evaluated by analyzing the directionality of play invitations. Our data showed that chimpanzees invited peers to play more often than non-peers, thus suggesting a preference to engage in play with matched individuals (Prediction 1 supported). Accordingly, other studies, carried out both in captivity and in the wild, demonstrated that immature chimpanzees tend to play with partners who are closest to themselves in age; for juveniles playing with infants might not have been challenging enough because of their limited motor skills and, on the other hand, for infants playing with juveniles might be too dangerous [41], [65].

Infant-infant dyads performed R&T more frequently than infant-juvenile and juvenile-juvenile dyads did (Prediction 1 supported); on the other hand, no difference was found for LR-play. Although, it was difficult to make direct comparisons between chimpanzee and human R&T due to the lack of quantitative data in children across different age stages [3], [16], some parallels seem to emerge. As in humans, the functions of R&T in chimpanzees shifted through the different developmental stages (Prediction 2 supported, see Table 3). In infancy, R&T seemed to have a role in socialization and in developing motor and psychological skills [3], [4]. In juveniles, R&T begins to include competitive elements that will be used by animals to establish social dominance relationships [16], [40], [66]. In humans, up to around 11 years, most evidence suggests that the great majority of R&T is purely playful, and that when play fighting does turn into real fighting, this is due to a lack of social skills and not the conscious manipulation that characterizes adolescents' R&T [67], [68]. Accordingly, Pellegrini [38] found that adolescent R&T was positively correlated with aggression and negatively correlated with social preference, thus suggesting that R&T could be a sort of training to acquire information on partner's skills; this information will be useful in the future to gain an advantage during real fights.

The asymmetry index of social play sessions seems not to be affected by the size and age of the players; however, when focusing on the matched dyads we found an interesting result. Social play between infants seems to be characterized by a higher degree of asymmetry compared to social play between juveniles (Prediction 1 supported). As play in juveniles is more competitive than in infants, the former have to restrain themselves in order to maintain as much as possible a symmetrical session and to limit the risk of escalation into serious fighting [8], [69]. Such an interpretation agrees with data on human youngsters, whose play bouts can turn into overt aggression [35], [38] (Prediction 2 supported). The unbalanced play sessions between infants may reflect different degrees of maturity in motor skills and/or the lack of capacity to fine-tune sessions due to difficulties in performing self-handicapping (a playmate, independently from his/her age, puts him/herself into unnecessary disadvantageous positions or situations) [3], [9] and motor inhibition [70]. This conclusion is supported by the lower complexity of infant play sessions characterized by few motor patterns which are highly repeated (see Fig. 4). In this view, the simpler R&T performed by infants could be a sort of training to assemble a more complex and sophisticated form of R&T typical of juvenility (play for play itself) (Prediction 1 supported). When playing with infants, juveniles have to self-handicap, promote reciprocal role-taking, and limit the number of types of patterns used. This capacity, also known as high-level response inhibition, relies on control mechanisms in anterior (in particular pre­frontal) cortical areas [70], [71]. This explains the lack of difference in play complexity recorded between I-I and I-J dyads. The higher complexity level recorded in juvenile dyads suggests that juvenile play is characterized by a greater number of innovative and unpredictable elements, thus indicating the development of novel control mechanisms in the cerebral cortex [70], [72]. The experience gained by switching between different play patterns may improve behavioral flexibility in chimpanzees in order to cope with unexpected situations (the training for the unexpected theory, [73]). Some studies on humans correspond to our data; the ability of children to switch activities during R&T was correlated with their capacity to face new social challenges [38], [74] (Prediction 2 supported).

Considering playful signals, infants and juveniles performed facial expressions with comparable frequency to maintain a playful interaction, thus indicating that there is no quantitative variation in the use of the two facial signals relating to the age of the performer. Moreover, no difference was found in the use of the two variants (PF and FPF) of play signals according to the age-phase. If we ended our analysis here, we should have to affirm that the Prediction 3a is not supported. However, the use of playful expressions varied according to the asymmetry of the session in infants, and to the receiver identity in juveniles (Prediction 3a partially supported). In infants, whose play sessions were the most unbalanced, we found a positive correlation between the frequency of playful facial displays and the degree of asymmetry characterizing each single session. In juveniles, we found that most of the facial signals were directed towards other juveniles. This result is not surprising if we consider the high complexity and competition levels characterizing chimpanzee juvenile play. Probably, when play becomes more competitive, as occurs in juvenile chimpanzees and humans [38], [67], [68], there needs to be clearer signaling to maintain the session and to avoid it turning into overt aggression [75]. Therefore, like laughter in humans (Duchenne laughter: [53], [55], [57], [76]), the playful expressions in chimpanzees (at the different age phases) seem to have a role in advertising cooperative dispositions and intentions thus increasing the likelihood of engaging in solid social relationships (Prediction 3a partially supported, see Table 3).

The presence of play faces during solitary play both in infant and juvenile chimpanzees indicates that, like in humans, playful facial displays can be an expression of an emotional state [57] (Prediction 3b supported), thus suggesting that infants of both species have the capacity for self-reflection or self-awareness (the precursors to more complex forms of social cognition, [44]). Recently, Pellis and Pellis [77] demonstrated that the role of play signals in self-regulating emotional state is also present in spider monkeys. Yet, there are some greater cognitive overlap and developmental similarities between *Pan* and *Homo* than between either and other primate species. This makes clear why the *Pan* genus offers special insights into the *Homo* genus in relation to some general aspects of play that are true for all species that play irrespective of their phylogenetic relationships [78].

In conclusion, like in humans, play in immature chimpanzees shows a number of changes, both quantitative and qualitative, across the ontogenetic pathway from infancy to juvenility, thus suggesting that chimpanzee play can have different functions according to the developmental stages of animals. This appears to be valid also for playful facial displays which, both in humans and chimpanzees, seem to function in modulating/enhancing social interactions and in expressing private emotions.

## Materials and Methods

### Ethics statement

This study was approved by University of Pisa (Animal Care and Use board). Since the study was purely observational the committee waived the need for a permit. The study was conducted with no manipulation of animals.

### The study species

The chimpanzee (*Pan troglodytes*) and bonobo (*Pan paniscus*) are the closest living relatives to humans [79]. These great apes share many basic features with humans. Both have a high level of behavioral flexibility and individuals aggregating into cohesive multimale-multifemale societies [80]. Chimpanzees live in communities, whose members form temporary parties that vary in size and composition [81]. The species is characterized by male philopatry and female dispersal, with females leaving their natal groups after reaching sexual maturity.

Similar to humans, a close bond with the mother characterizes behavioral development in chimpanzees that lasts until well after weaning. They have a long transition to independence [45]. Chimpanzee infants are in constant contact with their mother until about sixteen weeks. At this age, they remain out of contact (less than 5 m) from the mothers for few minutes per bout. Mothers continue to nurse and carry their offspring for about 4–5 years [80]. One-year-old infant chimpanzees begin to play with other infants often leaving their mothers for more than 10 m for several minutes [82]. Older siblings, when present, are the primary source of social interactions, but social contacts also include unrelated peers [80].

In this paper, we followed the age categories provided by Sugiyama [83] and classified immature subjects as follows: infants (I) from 0 to 3 years and juveniles (J) from 4 to 7 years (see Table 1 for age classification and sibling relation).

### The study groups

The Beauval colony lived in an enclosure composed by indoor and outdoor facilities of about 200 m^2^ and 2000 m^2^, respectively. The indoor facility was formed by two large enclosures that were placed in a glasshouse. The group received abundant food (vegetables, fresh fruits, nuts, grains, and yogurt) at 9.00 a.m., 2.00 p.m., and 4.30 p.m.

The Amersfoort colony was hosted in an enclosure made of indoor and outdoor facilities of about 80 m^2^ and 400 m^2^, respectively. The chimpanzees were fed three times a day (11.00 a.m., 1.00 p.m. and 3.00 p.m.) with pellets, vegetables, fruits, rice and nuts, that were scattered on the ground.

Both Beauval and Amersfoort enclosures were equipped with trunks, lianas, ropes, and platforms so the chimpanzees could move freely in all three dimensions.

### Data collection

Observations took place over a 6-hour period, 6 days per week (also covering the feeding-times) from October 2001 to February 2002 for the Beauval colony (for a total of 90 days) and from May to October 2004 for the Amersfoort colony (for a total of 91 days). Before systematic data were collected, the four observers underwent an 80-hour training period to become skilled in animal identification and behavioral pattern distinction. Training was over when the percentage agreement on animal and behavior recognition reached 95% among the observers [84] and when the Cohen's kappa was higher than 0.70 [85].

The authors with the two assistants were able to collect all playful interactions (see Table 2 for the behavioral item definition) by focal animal sampling method. All individuals were observed for exactly the same number of hours: 31 hrs of observation for the Beauval colony and 35 hrs of observation for the Amersfoort colony.

A solitary play session started when a lone individual performed a play pattern (see Table 2). If the bout started again after a delay of 10 s it was counted as a new play session.

A social play session was deemed to begin when one partner directed any playful behavior towards a playmate and ended when the participants stopped their activities or one of them moved away [58]. If the bout started again after a delay of 10 s it was counted as a new play session. For both social and solitary play sessions we recorded: i) playmate identity, ii) play patterns and their chronological sequence iii) context (circumstance in which play took place, e.g. feeding, sexual). For an accurate description of social play patterns see Table 2. Social play patterns were classified as unidirectional, when it is possible to distinguish an actor and a receiver (PBIT, PRE, PRUN, PSL, TK, PPS, PBR, PRCO, PST) and bidirectional, when the playmates are both actor and receiver (PIRO, ACP, SO).

Moreover, within social play we distinguished between locomotor-rotational (LR-play) and rough and tumble play (R&T). When a play session was characterized by the absence of any kind of physical contact, that session was labeled as LR- play [1], [86].

We also collected data on play invitations. A play invitation occurred when an animal approached a potential playmate, interacted with him/her by one of the play pattern considered (Table 2), and then ran away. Play invitations were labeled as PINV and PINV* according to the presence or absence of play sessions following the invitation.

For each play session, we recorded two variants of the playful facial displays: Play Face (PF, mouth is opened with only lower teeth exposed) and Full Play Face (FPF, mouth is opened with upper and lower teeth exposed) [13], [42], [78], [87]. For each playful facial display performed by the animals, we registered signaler and receiver identity (directionality) and the exact chronological sequence of the visual signals (strategic use of the signal, e.g. to initiate or maintain a play session).

### Data analysis

Data analysis focused on the 15 immature individuals (7 juveniles and 8 infants). Due to the non-normality of data and the small sample size (N = 15) nonparametric statistical tests were applied to the analyses performed at the individual level [88]. We made use of exact tests according to the threshold values suggested by Mundry & Fischer [89]. Non-parametric statistics was performed by using SPSS 12.0. The Wilcoxon matched-pair signed-ranks test (corrected for ties) was used for comparing i) the frequency of social and solitary play and ii) play signal directionality as a function of the age of playmates. The U-Mann Whitney test was used to contrast the level of playful facial displays (PF and FPF) performed by infants and juveniles.

When performing dyadic comparisons we used randomization procedures to avoid pseudo-replication due to non-independence of data (the same individual is included in more than one dyad, therefore dyads are not independent data-points). Specifically, randomization tests were employed with a number of 10,000 permutations using resampling procedures. In order to evaluate whether age significantly affected play distribution we analyzed data at the dyadic level (Infant-Infant, I-I; Infant-Juvenile, I-J; Juvenile-Juvenile, J-J) by applying the ANOVA randomization test [90]. Randomization post-hoc tests were used to determine which pairs of age combinations significantly differed. The two-independent randomization test was employed to assess whether possible differences existed between the Asymmetry Index (AI) of matched (I−I+J−J) and mismatched dyads (I-J). The correlation via randomization was used to assess possible correlations between play and grooming and play and agonistic contacts. All the dyadic analyses were performed by using Resampling Procedures 1.3 by David C. Howell (freeware).

All the analyses were two-tailed and the level of significance was set at 5%; however, trends (p<0.1) were also discussed.
